# General Pathology, as Conducive to the Establishment of Rational Principles for the Diagnosis and Treatment of Disease

**Published:** 1853-01

**Authors:** 


*General Pathology*, as conducive to the Establishment of Rational Principles for the Diagnosis and Treatment of Disease; a course of Lectures delivered at St. Thomas's Hospital, during the summer Session of 1850; by JOHN SIMON, F.R.S. One of the Surgical Staff of that Hospital, and Officer of Health to the city of London. Pp. 212, Philadelphia, Blanchard & Lea.

THE small work with the above title, may be classed among those of the many medical books recently published, worthy of a place in a well selected medical library. In expressing this opinion we do not wish to be understood as recommending these lectures of Dr. Simon as a standard work on general pathology. This branch of medical science has made too many advances and embraces too many facts to admit of being treated of in a single volume, like the one before us, of two hundred pages.

Practitioners and students, who, for want of time cannot study more elaborate works, will find in these lectures all the more important facts and principles of pathology treated of, in a manner interesting to the reader and creditable to the author.

In his introductory remarks, Dr. Simon, after giving a definition of Pathology, shows, most conclusively, that no real advancement can be made in this important branch, only so far as the facts and conclusions are based upon anatomical, physiological, chemical and clinical observations, since the symptoms and products of disease must be determined by types of healthy structure and function.

"Blood in disease," is the subject dwelt upon at some length in the second lecture. It is evident that Dr. Simon, very properly, as we think, is inclined to attach more importance to the character and condition of this fluid than most other writers upon the same subject. According to our author, Blood disease may result either from ingestion of morbid products, or from the retention and accumulation in the blood of matters which ought to be excreted. The most marked instances in which the blood becomes morbid from ingesta are those of poisoning by the introduction into the blood of substances which, from their nature or constitution, tend to destroy or impair the vital functions. It is evident also that the constituents of food might, under certain circumstances, such as in defective action in the assimilative organs, pass into the blood so imperfectly elaborated as to give rise to disease; so, too, pus globules and the germs of cancerous cells find their way into the circulating current, giving rise, in various parts, to the formation of abscess or the deposition of cancerous matter.

Blood may become diseased, also, from the accumulation and retention of excrementitious matters, such as should have been thrown out of the body by the excreting organs, such as the skin, liver, kidneys, &c.

In view of the fact that in the South and West, this accumulation of hydro-carbonaceous substances is favored by the high temperature and moist condition of an atmosphere more or less impregnated with carburetted hydrogen and other gases, the products of vegetable and animal decomposition, it is a question of interest and importance if, or not, this condition of the blood has an influence in modifying and producing periodic fevers. The color of the skin, conjunctiva, and even of the blood itself, would seem to indicate that the accumulation of carbon in the form, perhaps, of coloring matter, is one of the prominent pathological changes in this class of diseases. That such an accumulation is one of the causes of periodic fevers is evident also from the good effect of remedial agents, which, by exciting to action the liver, skin, kidneys, and other excreting organs, favor the excretion of hydro-carbonaceous matters.

Dr. Simon is fully up with the present time in his knowledge, not only of that branch upon which he professes to write, but also with the present advanced state of the science in general; hence, we can with confidence recommend the work before us as one of the best that has yet appeared on the subject. H.

